# Prevalence of Symptomatic Gallbladder Disease After Bariatric Surgery: A Literature Review

**DOI:** 10.7759/cureus.37777

**Published:** 2023-04-18

**Authors:** Isaac Alsallamin, Deema Chakhachiro, Ameed Bawwab, Monther Nassar, Afnan Alsallamin

**Affiliations:** 1 Internal Medicine, Case Western Reserve University School of Medicine, Cleveland, USA; 2 General Medicine, University Hospitals Cleveland Medical Center, Cleveland, USA; 3 Internal Medicine, Northeast Ohio Medical University, Cleveland, USA; 4 Internal Medicine, St. Vincent Charity Medical Center, Cleveland, USA; 5 Internal Medicine, University Hospitals Cleveland Medical Center, Cleveland, USA

**Keywords:** gallstone cholecystitis, prevalence rate, weight loss and obesity, obesity, bariatric surgery complications, gastric sleeve surgery, gallbladder disorders, gallbladder stones

## Abstract

Introduction

Gallbladder disease (GBD) encompasses several medical conditions, including gallbladder stone formation, biliary colic, and cholecystitis. These conditions may arise following bariatric surgery, including bypass or laparoscopic sleeve gastrectomy (LSG). The development of GBD after surgery may be attributed to various factors, including the formation of stones shortly after the procedure, the exacerbation of existing stones due to the surgery, or inflammation of the gallbladder. Rapid weight loss after surgery has also been proposed as a contributing factor.

Methodology

This observational study consisted of a review of retrospective hospital patient medical records of 350 adult participants who underwent LSG, with 177 participants included in the study after excluding those with cholecystectomy or GBD prior to surgery. The participants were followed for a median of two years, during which we recorded any hospitalizations, emergency department visits, clinic visits, and incidents of cholecystectomy or abdominal pain due to GBD. The participants were grouped into two: those with GBD and those without GBD after bariatric surgery, and quantitative data were summarized using mean and standard deviations. The data were analyzed using IBM SPSS Statistics for Windows, Version 20.0. (IBM Corp. Released 2020. IBM SPSS Statistics for Windows, Version 27.0. Armonk, NY: IBM Corp), with a statistical significance of P<0.05.

Results

In our retrospective study of 177 patients who underwent LSG, the incidence of GBD after bariatric surgery was 4.5%. Most patients with GBD after bariatric surgery were White, but this difference was not statistically significant. Patients with type 2 diabetes had a higher incidence of GBD after bariatric surgery than those without diabetes (8.3% vs. 3.6%, P=0.355). Patients with HTN had a lower incidence of GBD after bariatric surgery than those without HTN (1.1% vs. 8.2%, P=0.032). Anti-hyperglycemia medication use did not significantly increase the risk of GBD after bariatric surgery (7.5% vs. 3.8%, P=0.389). None of the patients on weight loss medication developed GBD after bariatric surgery, compared to 5% of patients who did not take weight loss medication. Our sub-data analysis showed that patients who developed GBD after bariatric surgery had a high BMI (above 40 kg/m2) before surgery, which decreased to 35 kg/m2 and below 30 kg/m2 at six months and 12 months post-surgery, respectively.

Conclusions

Our findings demonstrate that the prevalence of GBD after LSG is low and comparable to the general population without LSG. Thus, LSG does not increase the risk of GBD. We found that rapid weight loss after LSG is a significant risk factor for GBD. These findings suggest that patients who undergo LSG should be informed of the risks of GBD and undergo careful screening before surgery to detect any pre-existing gallbladder issues. Overall, our study highlights the importance of continued research into the factors associated with GBD after bariatric surgery and the need for standardized prophylactic measures to prevent this potentially serious complication.

## Introduction

Bariatric surgery, also known as weight loss and metabolic surgery, is an effective method for treating obesity and several comorbidities, including type 2 diabetes (T2D), HTN, sleep apnea, and high blood cholesterol in obese patients. The operation is generally safe in expert hands, with lower complication rates than other abdominal operations [1,2]. Laparoscopic sleeve gastrectomy (LSG) has become the most common bariatric surgery because it is simple, safe, and effective in treating obesity [1,2]. However, patients may develop gallbladder disease (GBD) after bariatric surgery, which is a rare complication.

GBD is a broad diagnosis that includes several conditions, such as asymptomatic cholelithiasis, biliary colic, acute calculous cholecystitis, chronic cholecystitis, choledocholithiasis, and Mirizzi syndrome, among others. These pathologies are often associated with the formation of gallstones that obstruct different areas of the bile drainage system. While asymptomatic gallstones can be challenging to diagnose, symptomatic GBD is often treated with cholecystectomy and other modalities to relieve obstructive symptoms.

According to the Third National Health and Nutrition Examination Survey in 2021, the estimated national prevalence of GBD in the United States is 7.9% in men and 16.6% in women [3]. The prevalence of GBD is even higher in obese individuals, with a relative risk ratio of 3.75 to 4.57 compared to non-obese individuals [1]. Studies have shown that the risk of symptomatic gallstone disease increases by 7% for every 1-kg/m2 increase in measured BMI [1,3,4]. Nonmodifiable risk factors for gallstone formation include age, sex (female), ethnicity, genetic susceptibility, and family history, while modifiable risk factors include pregnancy, dyslipidemia, obesity, rapid weight loss, metabolic syndrome, medications, gallbladder stasis, terminal ileal disease, cirrhosis, and hyperbilirubinemia [5].

The incidence of gallbladder stone formation after bariatric surgery varies. Some investigations have reported that 3% to 22% of bariatric patients develop gallstones within 12 months after surgery, and 8% to 30% develop them within 24 months after surgery [6,7]. Nearly 4.7% to 12% of bariatric patients require gallbladder removal during active weight loss. Obesity is a risk factor, but conversely, rapid weight loss can also increase the likelihood of gallstone formation. One study showed that the general population’s traditional risk factors for gallstone formation are not predictive of symptomatic gallstone formation after bariatric surgery. Weight loss of more than 25% of the original weight was the only postoperative factor that helps select patients for postoperative ultrasound surveillance and subsequent cholecystectomy once gallstones were identified [1,3,4].

In our clinical experience working with bariatric patients, we have observed several cases of readmission to the hospital due to abdominal pain stemming from various causes, including gastroesophageal reflux disease, epigastric pain related to biliary colic, gallbladder stones, and surgical complications such as constipation, partial bowel obstruction, or gastric leak. These observations prompted us to question the prevalence of GBD following bariatric surgery and whether it is a common occurrence. Consequently, this study aims to investigate the prevalence of GBD after LSG at St. Vincent Charity Medical Centre (SVCMC) and explore the potential relationship between GBD and rapid weight loss.

## Materials and methods

We retrospectively reviewed the medical records of 350 patients who underwent LSG at the Centre for Bariatric Surgery at SVCMC from 2017 to 2022. The study included patients referred for weight loss surgery due to morbid obesity, specifically for LSG. This research was approved by the Institutional Review Board for Research and Ethics at SVCMC (approval no. 554).

This study excluded patients younger than 18 years of age, pregnant patients, those with a history of GBD before surgery, cholecystectomy before surgery, documented gallstones, or previous history of biliary colic. After applying the exclusion criteria, we ended up with 177 patients, and four patients were lost to follow-up.

The inclusion criteria include those who developed GBD after surgery, those who presented with abdominal pain due to biliary colic that was confirmed by ultrasound, CT scan, or hepatobiliary iminodiacetic acid (HIDA) scan, or those who had a cholecystectomy after LSG.

We calculated the prevalence of GBD after LSG and found that eight patients developed the disease out of 177 patients (4.5%). We then compared the two groups of participants, those with GBD (4.5%) and those without GBD (93%), based on medication use and change in BMI before and after surgery.

We reviewed patient medical records for documented GBD, symptomatic gallstones, cholecystitis, or abdominal pain due to GBD after LSG surgery at three, six, nine, and 12 months after the surgery. The diagnosis of GBD was documented and confirmed with positive findings on ultrasound, HIDA scan, or CT scan for new GBD and diagnosis on discharge with biliary colic.

We also measured the patients' BMI, glycated hemoglobin (HbA1c), and confounders such as the use of weight loss medications and comorbidities like T2D and HTN and related medications before and after the LSG procedure, regardless of the indication for endoscopy. We collected demographic data (e.g., age, race, sex, medication use, and comorbidities) and evaluated GBD at three, six, nine, and 12 months. Data were collected using Microsoft Excel (Microsoft Corporation, Redmond, WA) and analyzed using IBM SPSS Statistics for Windows, Version 20.0 (IBM Corp. Released 2020. IBM SPSS Statistics for Windows, Version 27.0. Armonk, NY: IBM Corp).

Statistical analysis

For categorical data, summary statistics are counts and percentages, and the comparisons between groups were made using Fisher’s exact test or the chi-square test of proportions for contingency table data, as appropriate. The quantitative data are summarized using the mean and standard deviation, maximum, minimum, median, and all quartiles, as well as the number of data points. As the sample size was small for patients with GBD present after bariatric surgery, we used the nonparametric Mann-Whitney U test for independent groups to perform statistical comparisons between groups. For hypothesis tests, the statistical significance was P<0.05.

## Results

Our retrospective study included 177 patients who underwent LSG. Of these, 17.7% were male, and 82.3% were female. The incidence of GBD after bariatric surgery was 3.2% among male patients and 4.9% among female patients. White patients comprised most of those with GBD after bariatric surgery, but this difference was not statistically significant (P=0.86; Table 1).

**Table 1:** Demographic distribution GBD: gallbladder disease

Demographic data	GBD absent	GBD present
Male patients		
Count	30	1
% of males	96.80%	3.20%
% with GBD after bariatric surgery	18.00%	12.50%
Female patients		
Count	137	7
% of females	95.10%	4.90%
% with GBD after bariatric surgery	82.00%	87.50%
Black race		
Count	40	2
% of black race	95.20%	4.80%
% with GBD after bariatric surgery	24.20%	25.00%
Caucasian or White race		
Count	121	6
% of Caucasian or White race	95.20%	4.80%
% with GBD after bariatric surgery	72.10%	75.00%
Other races		
Count	6	0
% of non-Black, non-White race	100.00%	0.00%
% with GBD after bariatric surgery	3.60%	0.00%

By reviewing the characteristics of our eight patients with GBD after LSG, we found out that four cases presented with biliary colic, and five cases were diagnosed with cholecystitis. Eight patients underwent cholecystectomy, and two patients presented with biliary colic underwent cholecystectomy without a clear diagnosis of the type of GBD. For the time frame, one case developed GBD after 20 days from the LSG, four cases within six months, two cases after 18 months, and one case after two years. Three patients aged 20-30 years, four patients 39- 45 years, and one patient above the age of 70 at the time of the diagnosis with GBD. One patient is Black, and seven patients are White. Two cases were excluded because they had cholecystectomy before LSG. No atypical cases of GBD were reported except for one case involving a 44-year-old young Black woman with biliary ectasia that was seen after 17 months of bariatric surgery and presented with abdominal pain. In our study, 8.3% of T2D patients developed GBD after bariatric surgery, compared to 3.6% without T2D (Table 2).

**Table 2:** Diabetic patients who developed GBD after surgery GBD: gallbladder disease, T2D: type 2 diabetes

T2D data	GBD absent	GBD present
T2D Patients		
Count	33	3
% with T2D	91.70%	8.30%
% with GBD after bariatric surgery	20.00%	37.50%
T2D medications		
% taking anti-T2D medication, insulin or sulfonylureas, metformin	92.50%	7.50%
% with GBD after bariatric surgery	22.40%	37.50%

The use of anti-hyperglycemia medication did not significantly increase the risk of GBD after bariatric surgery (7.5% vs. 3.8%, P=0.389). Conversely, patients with HTN had a lower incidence of GBD after bariatric surgery than those without HTN (1.1% vs. 8.2%, P=0.032; Table 3).

**Table 3:** Patients with HTN who developed GBD GBD: gallbladder disease, HTN: hypertension

HTN data	GBD absent	GBD present
Count	87	1
% with HTN	98.90%	1.10%
% with GBD after bariatric surgery	52.70%	12.50%

In addition, none of the patients on weight-loss medication developed GBD after bariatric surgery, compared to 5% of the patients not on weight-loss medication (Table 4).

**Table 4:** Patients on medications for weight loss and cholesterol reduction who developed GBD after surgery ^a^Weight loss medications include phentermine-topiramate orlistat, bupropion, topiramate, and diethylpropion

Medication data	GBD absent	GBD present
Weight loss medications		
Count	13	0
% taking weight loss medications^a^	100.00%	0.00%
% with GBD after bariatric surgery	7.90%	0.00%
GLP-1 (liraglutide/Saxenda)		
Count	0	6
% taking GLP-1	0.00%	100.00%
% with GBD after bariatric surgery	0.00%	3.60%
Cholesterol-lowering agents		
Count	36	0
% taking cholesterol-lowering agents, statins	100.00%	0.00%
% with GBD after bariatric surgery	21.80%	0.00%

Finally, our sub-data analysis revealed that patients who developed GBD after bariatric surgery had a high BMI (above 40 kg/m2) before surgery, which decreased to 35 kg/m2 and below 30 kg/m2 at six months and 12 months post-surgery, respectively. We found no significant differences in the distribution of age, BMI before and after surgery, or HbA1c levels between the two groups (Table 5).

**Table 5:** GBD after bariatric surgery in relation to change in BMI after the surgery BMI: body mass index, GBD: gallbladder disease, HbA1c: glycated hemoglobin, SD: standard deviation

Presence of GBD after bariatric surgery	Age (years)	BMI before surgery (kg/m^2^)	BMI six months after surgery (kg/m^2^)	BMI 9-12 months after surgery (kg/m^2^)	HbA1c before surgery
N valid/missing	8/0	8/0	5/3	5/3	8/0
Mean	40.13	39.13	35.8	31.8	6.2
Median	41	37.5	34	32	5.65
Std. deviation	15.986	6.058	5.404	3.347	1.9086
25 percentiles	26.25	35	32	28.5	5.15
50 percentiles	41	37.5	34	32	5.65
75 percentiles	44.75	42.5	40.5	35	6.425

## Discussion

The association between bariatric surgery and GBD has been well-documented in recent years. Rapid weight loss after Roux-en-Y gastric bypass (RYGB) is a strong risk factor for GBD [8]. However, with the emergence of LSG as the first choice of treatment for obesity and bariatric surgery, postoperative complications and associations are being investigated to learn more about its safety. Our study examined the incidence of GBD in 177 patients up to 24 months after LSG. The incidence of GBD after LSG was 4.6%, which is similar to previous studies that showed rates ranging from 3.3% to 9.5% [9-11].

The development of GBD following bariatric surgery can be attributed to several factors. Some patients may have had preexisting asymptomatic GBD before surgery, which could be exacerbated by the procedure itself or progress along the natural course of the disease, irrespective of surgery [3,6,8]. The fact that the patient has undergone surgery may also prompt more thorough investigations whenever they present with abdominal pain, potentially leading to the detection of previously asymptomatic GBD that is then mistakenly blamed for the pain. Furthermore, metabolic changes post-surgery may alter bile acid production and contribute to the formation of sludge, which can result in the development of new stones or exacerbation of existing GBD [3,6,8].

Prophylactic cholecystectomy, intraoperative ultrasound for gallstone detection (with concomitant cholecystectomy), postoperative ursodeoxycholic acid, and regular ultrasound surveillance have been used to prevent GBD after bariatric surgery [1,3,4]. However, none of these prophylaxis measures have been accepted as standard practice, and conservative approaches have been the mainstay of treatment for asymptomatic cholecystitis, as further research is needed to support other modalities [9]. Although dietary adjustments can aid in preventing gallstone formation [1,9], more studies are needed to confirm their effectiveness.

It has been established that obesity is a risk factor for GBD [12,13], and our study found that rapid weight loss after LSG is also a significant risk factor for GBD, which is consistent with prior studies on RYGB [3,8,14]. Interestingly, our study found that HTN was negatively associated with GBD after LSG, as only 1.1% of HTN patients developed GBD compared to 8.2% of patients without HTN (p=0.032). Further research is needed to elucidate the underlying mechanism for this phenomenon, which may be attributed to lifestyle choices that HTN patients make or cholesterol-lowering medications. On the other hand, none of the other factors we examined, including T2D, use of cholesterol-lowering agents, and use of anti-hyperglycemia medications, such as sulfonylureas, insulin, or metformin, were statistically significant in their association with GBD after sleeve gastrectomy.

Furthermore, the risk of GBD development may be heightened by using certain medications, which can impact the disease through several theoretical mechanisms. Some medications may affect lipoprotein uptake by hepatocytes or inhibit the activity of acyl coenzyme A-cholesterol acyltransferase, leading to bile production with sludge [5,14,15]. Other medications may promote the precipitation of bile salt formations, while less common mechanisms include hypersensitivity cholecystitis [5,14,15].

There are limitations to our study that must be acknowledged. First, our study has a small sample size of 177 patients, indicating the need for larger studies to confirm our findings. Second, the effect of multivariate factors, such as exercise and diet control, changes in medication doses, frequency or compliance during the follow-up period, and some medications, may influence the weight changes or the development of GBD, which, in turn, may influence the result of this study and will be difficult to eliminate. Third, there were patients that were lost to follow-up and not included in our study, and we could not report the use of ursodeoxycholic acid or other agents that may decrease the formation of gallstones. Fourth, we did not report the severity of the disease, which can affect the approach and contaminate our sample. Finally, as a retrospective study, other factors that were not controlled for may be related to our findings. Therefore, further studies are needed to confirm our findings and elucidate the underlying mechanisms for our observed associations.

## Conclusions

This study aimed to investigate the prevalence of GBD after LSG and the potential relationship between GBD and rapid weight loss. Our findings demonstrate that the prevalence of GBD after LSG is low and comparable to the general population without LSG. Thus, LSG may not increase the risk of GBD, which is an important finding. We found that rapid weight loss after sleeve gastrectomy is a significant risk factor for GBD after LSG. These findings suggest that patients who undergo LSG should be informed of the risks of GBD and undergo careful screening before surgery to detect any pre-existing gallbladder issues. Overall, our study highlights the importance of continued research into the factors associated with GBD after bariatric surgery and the need for standardized prophylactic measures to prevent this potentially serious complication.

## References

[1] Climaco K, Ahnfeldt E (2021). Laparoscopic vertical sleeve gastrectomy. Surg Clin North Am.

[2] Himika D, Katsnelson V, Alsallamin I, Bawwab A, Chakhachiro D (2022). The effect of laparoscopic sleeve gastrectomy on symptoms of gastroesophageal reflux disease. Cureus.

[3] Li VK, Pulido N, Fajnwaks P, Szomstein S, Rosenthal R, Martinez-Duartez P (2009). Predictors of gallstone formation after bariatric surgery: a multivariate analysis of risk factors comparing gastric bypass, gastric banding, and sleeve gastrectomy. Surg Endosc.

[4] Unalp-Arida A, Ruhl CE (2022). The burden of gallstone disease in the United States population. medRxiv.

[5] Lam R, Zakko A, Petrov JC, Kumar P, Duffy AJ, Muniraj T (2021). Gallbladder disorders: a comprehensive review. Dis Mon.

[6] Miller K, Hell E, Lang B, Lengauer E (2003). Gallstone formation prophylaxis after gastric restrictive procedures for weight loss: a randomized double-blind placebo-controlled trial. Ann Surg.

[7] Haal S, Guman MSS, Boerlage TCC (2021). Ursodeoxycholic acid for the prevention of symptomatic gallstone disease after bariatric surgery (UPGRADE): a multicentre, double-blind, randomised, placebo-controlled superiority trial. Lancet Gastroenterol Hepatol.

[8] Iglézias Brandão de Oliveira C, Adami Chaim E, da Silva BB (2003). Impact of rapid weight reduction on risk of cholelithiasis after bariatric surgery. Obes Surg.

[9] Morais M, Faria G, Preto J, Costa-Maia J (2016). Gallstones and bariatric surgery: to treat or not to treat?. World J Surg.

[10] Manatsathit W, Leelasinjaroen P, Al-Hamid H, Szpunar S, Hawasli A (2016). The incidence of cholelithiasis after sleeve gastrectomy and its association with weight loss: a two-centre retrospective cohort study. Int J Surg.

[11] Moon RC, Teixeira AF, DuCoin C, Varnadore S, Jawad MA (2014). Comparison of cholecystectomy cases after Roux-en-Y gastric bypass, sleeve gastrectomy, and gastric banding. Surg Obes Relat Dis.

[12] Cruz-Monserrate Z, Conwell DL, Krishna SG (2016). The impact of obesity on gallstone disease, acute pancreatitis, and pancreatic cancer. Gastroenterol Clin North Am.

[13] Stender S, Nordestgaard BG, Tybjaerg-Hansen A (2013). Elevated body mass index as a causal risk factor for symptomatic gallstone disease: a Mendelian randomization study. Hepatology.

[14] Haal S, Guman MS, Bruin S (2022). Risk factors for symptomatic gallstone disease and gallstone formation after bariatric surgery. Obes Surg.

[15] Michielsen PP, Fierens H, Van Maercke YM (1992). Drug-induced gallbladder disease. Incidence, aetiology and management. Drug Saf.

